# Temperature Measurement in WTE Boilers Using Suction Pyrometers

**DOI:** 10.3390/s131115633

**Published:** 2013-11-15

**Authors:** Fabio Rinaldi, Behzad Najafi

**Affiliations:** Department of Energy, Politecnico di Milano, Via Lambruschini 4, Milano 20156, Italy; E-Mail: behzad.najafi@polimi.it

**Keywords:** temperature measurement, waste-to-energy plants, uncertainty, suction pyrometers

## Abstract

The temperature of the flue-gas in the post combustion zone of a waste to energy (WTE) plant has to be maintained within a fairly narrow range of values, the minimum of which is prescribed by the European Waste Directive 2000/76/CE, whereas the maximum value must be such as to ensure the preservation of the materials and the energy efficiency of the plant. A high degree of accuracy in measuring and controlling the aforementioned temperature is therefore required. In almost the totality of WTE plants this measurement process is carried out by using practical industrial thermometers, such as bare thermocouples and infrared radiation (IR) pyrometers, even if affected by different physical contributions which can make the gas temperature measurements incorrect. The objective of this paper is to analyze errors and uncertainties that can arise when using a bare thermocouple or an IR pyrometer in a WTE plant and to provide a method for the *in situ* calibration of these industrial sensors through the use of suction pyrometers. The paper describes principle of operation, design, and uncertainty contributions of suction pyrometers, it also provides the best estimation of the flue-gas temperature in the post combustion zone of a WTE plant and the estimation of its expanded uncertainty.

## 1. Introduction

Sustainable and optimized energy conversion systems have become increasingly popular and sought after with the ever growing energy demand, high energy prices, reduction of fossil fuel resources, and increase of local and global atmospheric emissions [1–3]. Waste can be considered as a renewable energy source since it is related to all human activities; combustion of this never-ending fuel in municipal solid waste (MSW) incinerators is quite an old practice and can significantly contribute in reducing dependency on fossil fuel if the appropriate waste-to-energy (WTE) technology is applied [4–6]. Waste combustion treatment is widely applied throughout Europe, although it is still looked upon in a negative light and not fully accepted, especially by environmentalist associations and some local communities in most of the European Member States.

In order to prevent or to limit negative effects on the environment, as far as practicable, in particular emissions into the air and resulting pollution of soil, surface waters and groundwaters, and the resulting risks to human health [7,8], the European Parliament and the Council of the European Union has laid down stringent operational conditions and technical requirements, by fixing emission limit values for waste incineration plants. As regards the operative temperature control, the latest European Waste Directive [9] lays down that:

1. Flue-gas resulting from the combustion process has to be maintained, after the last injection of air to a temperature of at least 850 °C, as measured near the inner wall for two seconds. If hazardous wastes with a content of more than 1% of halogenated organic substances, expressed as chlorine, are incinerated, the temperature has to be guaranteed to a value of at least 1,100 °C for two seconds;
2. The auxiliary burners, that must be fitted to each incineration plant line, must activate automatically to prevent that the temperature of the combustion gas falls below 850 or 1,100 °C whatever the case may be.

From these brief considerations one may see that in order to respect the EU Directive and the study, inspection and conduction of WTE plants a high degree of accuracy in temperature readings is of paramount importance.

## 2. Waste to Energy Boiler Design

Design parameters and operating characteristics of a modern refuse-fired boiler depend on the refuse composition, which change depending on time and location [10–12]. The higher heating value (HHV) of waste ranges from 7,000 to 14,000 kJ/kg; this value is generally proportional to the industrialization of the country and depends mainly on plastics, paper and moisture content and on the degree of fuel preparation. There can be two different types of fuel: as-received in its unprepared state by just removing only large and non-combustible items or as refuse-derived fuel (RDF), where a selected part of the as-received refuse is shredded and fed into the furnace.

WTE boilers are usually single drum, top supported with natural circulation and are able to supply superheated steam to a vapor turbine, in order to produce electrical energy by means of an alternator. In some cases the boiler is designed to recover wasted heat for district heating purposes. Saturated pressure ranges usually between 4 and 8 MPa, whereas the maximum temperature of superheated steam is limited by the material resistance of the superheater.

The boiler can be ideally divided into three parts: a furnace in which waste combustion takes place, a radiant zone in which the combustion is completed and where the heat, from flue-gases to water, is mainly exchanged by radiation and a convection zone where heat is exchanged mostly by convection.

Combustion in the furnace takes place on a reciprocating combustion water or air-cooled grate, solid waste rolls along the stoker grate from the loading zone to the ash pit where the non-combustible part is eliminated. If RDF is used combustion takes place partly on a stocker and in part in suspension; a fluidized-circulating-bed or a bubbling fluidized-bed can also be utilized.

The furnace sides are fully water-cooled with spaced tubes or with tubes placed next to one another, the spaced tube configuration being the most common: tubes are connected using a welded continuous fin (membrane bar), thus building a monolithic wall called a membrane wall [13]. Each furnace is designed for a rather strict range of thermal load for which its volume depends both on the HHV and on the mass flow rate of the fuel.

**Figure 1.**
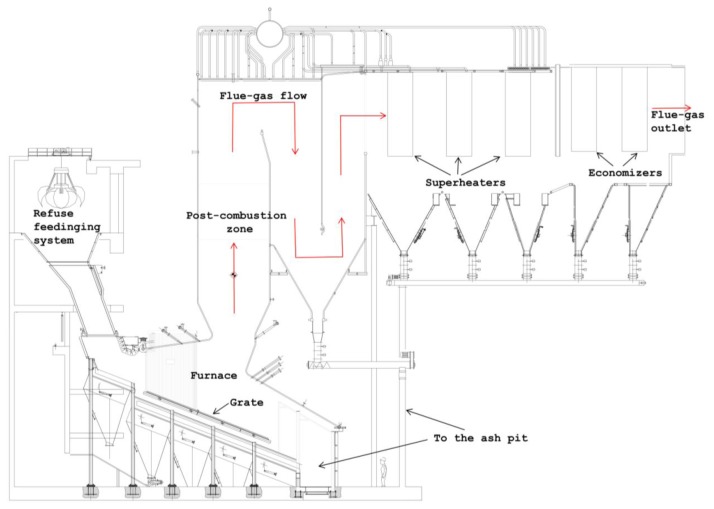
Typical waste to energy (WTE) boiler configuration.

As shown in Figure 1, the flue-gas leaving the furnace grate flows alternatively upward and downward through the open passageways of the radiant zone whose number, typically three, is dependent on the boiler configuration.

Flue-gases leaving the radiant zone then enter the horizontal convective zone; this part of the boiler typically includes a first evaporative section, two or more superheating sections followed by another evaporator and economizers. The superheater is generally placed in a zone where the maximum gas temperature is 600 °C (in order to guarantee stable operations over a long period of time) and it is subdivided into two or more sections, with intermediate regulation of temperature through the injection of feed water, to keep the pipes surface temperature under control.

Combustion products from waste incineration are very corrosive mainly because of the presence of chlorine compounds, the rate of tube metal loss due to corrosion increases as the metal temperature increases. For this reason the aforementioned furnace membrane walls and the radiant zone, where the flue-gas temperature are sufficiently high, must be protected. Moreover in the lower part of the radiant zone, the so-called post-combustion zone, heat absorption must be reduced to maintain adequate gas temperatures in all load-firing conditions. In the post combustion zone membrane walls are therefore overlaid by using silicon carbide refractory whereas the other membrane walls of the radiant zone are generally protected by using Inconel^®^ weld cladding [14].

Both the silicon carbide cast refractory and the Inconel^®^ weld overlay must have a proper rate of thermal conductivity to maintain the gas temperature at 850 °C for at least two seconds and to guarantee the effectiveness of the water-cooled surface that they are protecting. Maintaining a low membrane walls surface temperature is essential to increase their lifespan and to reduce wall fouling and maintenance costs.

## 3. Gas Temperature Measurement in Waste-to-Energy (WTE) Boilers

Temperature measurement of combustion gases at different locations within the boiler is important to both the boiler design and the operating plant engineers. Accurate gas temperature measurements can confirm design predictions and operating performance, allows working order in ideal temperature ranges, maximum plant energy efficiency, and increases the lifespan of both the materials and the components.

Bare thermocouples and radiation pyrometers are generally used in nearly all WTE plants. It is however the commonplace experience of plant operating engineers that temperature measurements given by these instruments differ from the real gas temperature, even by a number of degrees.

Whatever instrument is used for temperature readings, the temperature sensitive element of an industrial thermometer will stabilize at a level which balances the heat flow by convection between the element and the gas, against the heat flow by radiation and conduction. The element temperature can be determined by the simultaneous solution of three heat flow rates; the energetic balance per unit of time *t* of the thermal element is therefore:

(1) $$\frac{\partial U}{\partial t}=M\cdot c_{v}\frac{\partial T}{\partial t}=\rho \cdot V\cdot c_{v}\frac{\partial T}{\partial t}={\dot{Q}}_{cv}-{\dot{Q}}_{\text{rad}}-{\dot{Q}}_{cd}$$

where *U*, *c_v_*, ρ and *V* are the internal energy, the specific heat, the density and the volume of the thermal element, respectively. During transients, the element will lag behind any change in gas temperature, due to its thermal capacity, resulting in a response-rate error.

### 3.1. Errors Using a Bare Thermocouple

For the hot junction of a thermocouple, the equations for radiant, conductive, convective heat transfer and for response rate are well known [15–17].

In WTE applications thermocouples are inserted inside a volume where the hot flue-gas is surrounded by cold membrane walls, this is particularly true in the radiant zone of the boiler where the heat flux exchanged by radiation is dominant.

Any difference between thermocouple junction temperature *T_tc_* and gas stream total temperature *T_g_* is considered to be an error *E*. The following errors are evaluated considering a steady state condition, so that Equation (1) reduces to:

(2) $${\dot{Q}}_{cv}={\dot{Q}}_{\text{rad}}-{\dot{Q}}_{cd}$$

#### 3.1.1. Conduction Error

This error *E_cd_* can be calculated considering a thermocouple wire of a length *l*, immersed in a homogeneous flow of gas at constant temperature *T_g_* and immediately emerging into an environment with constant temperature *T_e_*. In stationary state conditions this error can be evaluated as:

(3) $$E_{cd}=T_{tc}-T_{g}=\frac{T_{e}-T_{g}}{\cos\;h\left[{\left(\frac{4h_{cv}}{kd}\right)}^{\frac{1}{2}}l\right]+{\left(\frac{h_{cv}}{k}\right)}^{\frac{3}{2}}{\left(\frac{d}{2}\right)}^{\frac{1}{2}}\sin\;\text{h}\left[{\left(\frac{4h_{cv}}{kd}\right)}^{\frac{1}{2}}l\right]}$$

The magnitude of *E_cd_* can be reduced by immersing the thermocouple in the gas flow with as great a length as possible, with a low thermal conductivity *k* and by increasing the heat transfer coefficient by convection *h_cv_*.

The heat transfer coefficient *h_cv_* between gas and thermocouple can be calculated by the relation between the Nusselt number and the Reynolds number, the following relations [18] are valid for combustion gas where Prandtl number is about 0.7:

(4) Nu=(0.44±0.06)(Re)0.5

(5) Nu=(0.085±0.009)(Re)0.674

Equation (4) refers to a thermocouple placed normal to the flow direction, whereas Equation (5) is for a thermocouple placed along the flow direction.

The gas velocity in a WTE plant generally varies between 0 and 10 m/s. As a consequence, *h_cv_* is between 0 and 180 W·m^−2^·K^−1^). These parameters have been calculated for a type K thermocouple with a diameter of 4 mm and placed in normal line to the gas flow direction.

**Figure 2.**
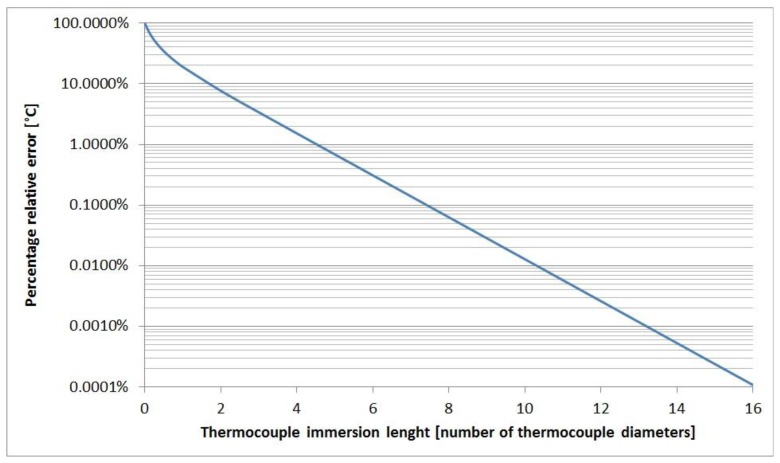
The relative percentage temperature error against thermocouple immersion length in diameters.

Even with temperature differences of more than 900 °C between gas and environment, the conduction error is less than 0.001 °C, if the length of the immersed part of the thermocouple inside the boiler is greater than 15 thermocouple diameters. Figure 2 shows the relative percentage temperature error, *E_cd_*/(*T_e_* − *T_g_*)·100, plotted against the thermocouple immersion length.

#### 3.1.2. Radiation-Convection Error

This error *E_cv-rd_* can be calculated neglecting the heat flux by conduction and considering both the convection and radiation Equations (6) and (7):

(6) $${\dot{Q}}_{cv}=A_{cv}h_{cv}(T_{g}-T_{tc})$$

(7) $${\dot{Q}}_{rd}=A_{rd}F_{tc-\text{wall}}\sigma \varepsilon (T_{tc}^{4}-T_{\text{wall}}^{4})$$

The radiation-convection error is therefore:

(8) $$E_{cv-rd}=(T_{g}-T_{tc})=\frac{A_{rd}F_{tc-\text{wall}}\sigma \varepsilon (T_{tc}^{4}-T_{\text{wall}}^{4})}{A_{cv}h_{cv}}$$

This error for a thermocouple inside an enclosure can be expressed as a simplified form of Equation (8), if the enclosure is greater compared to the thermocouple diameter, as it is in the WTE applications. In our particular case the radiation view factor *F_tc-wall_* equals 1 and the area available for radiation *A_rd_* can be considered equal to the area available for convection *A_cv_*; the expression of *E_cv-rd_* can be therefore written as:

(9) $$E_{cv-rd}=(T_{g}-T_{tc})=\frac{\sigma \varepsilon (T_{tc}^{4}-T_{\text{wall}}^{4})}{h_{cv}}$$

To reduce this error, thermocouple emissivity must be maintained as low as possible while *h_cv_* must be as high as possible.

Polished metal surfaces have a low emissivity [19] (ε ≤ 0.2) at temperatures below 250 °C but emissivity increases rapidly with temperature as well as by oxidation or deposition on the surface. In some case emissivity of thermocouple can easily and quickly reach 0.8.

The wall temperature can be predicted by the boiler designer as a function of the boiler thermal load and the boiler external conditions. In Equations (7)–(9) the wall temperature should be replaced by the mean radiating temperature of the thermocouple surrounding, the combustion gas is in fact laden with solid particles at temperature and emissivity which are different to that of both the gas and the wall.

**Figure 3.**
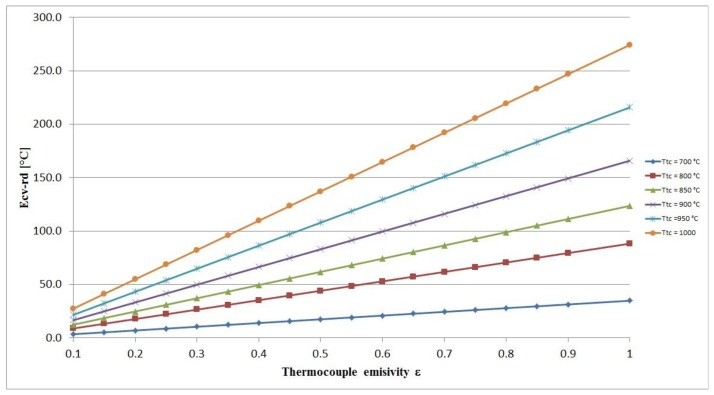
Radiation convection error estimated for a wall temperature of 600 °C.

Assuming the gas and environmental conditions in a zone of the incinerator plant, the numerical value of the radiation-convection error can be estimated by Equation (9). In Figure 3 the numerical estimation of the radiation-convection error is provided for a wall temperature of 600 °C and for different thermocouple temperatures.

#### 3.1.3. Transient Error

The temperature of a thermocouple junction will always lag behind any change in the temperature of the gas in which it is immersed. Heat must be transferred between the gas and the junction to accomplish a change in junction temperature. The rate of heat transfer depends on the values of *h_cv_*, on the area available for convection and on the difference between junction and gas temperature. Such a temperature difference must then exist if the junction temperature is immersed in a gas which temperature is changing with time. Its magnitude *E_t_* can be calculated from the measured rate of change of junction temperature, as shown in Equation (10):

(10) $$E_{t}=T_{g}-T_{tc}=\tau \frac{\partial T_{tc}}{\partial t}$$

The behavior of a thermocouple during a transient can be expressed in terms of its time constant, defined in Equation (11):

(11) $$\tau =\frac{\rho cV}{h_{cv}A_{cv}}$$

where ρ, *c* and *V* are the density, the specific heat and the volume of the thermocouple junction, respectively. A typical time constant value for a thermocouple utilized in a WTE boiler ranges between 30 and 300 s. Bare thermocouples placed in the post-combustion-zone of an incinerator plant are particularly subject to structural alterations by chemical corrosion, oxidation and fused salt depositions. Consequently their constant time can undergo many order variances during measurement time.

### 3.2. Errors Using Infrared Radiation (IR) Pyrometers

In many WTE boilers infrared radiation (IR) pyrometers are used for measuring the flue-gas temperature. The IR pyrometer is a measuring transducer, which receives the infrared radiation emitted by the measuring object itself and converts it into a standardized output signal. The radiation energy per time unit measured by the IR thermometer, for a selected wavelength λ, is called spectral radiance *L*:

(12) $$L_{\lambda ,m}=\varepsilon_{\lambda }L_{\lambda ,b}(T_{g})$$

The temperature is calculated by solving:

(13) $$L_{\lambda ,b}(T_{m})=\frac{\varepsilon_{\lambda }L_{\lambda ,b}(T_{g})}{\varepsilon_{i}}$$

Ideally the instrumental emissivity ε*_i_* has to be fixed equal to the emissivity ε_λ_ of the flue-gas, so that the measured temperature *T_m_* is the true gas temperature *T_g_* [20].

Despite the fact that many IR pyrometer manufacturers indicate that the selective temperature reading of certain gases is possible by means of narrow-band filters, these instruments are not designed to measure gas temperature. This is particularly true in a WTE boiler environment where the IR pyrometer collects radiation energy from a well-defined conical volume in front of it, the flue-gas in this zone is laden with solid particles and its composition is variable (carbon monoxide and dioxide, nitrogen, water vapor, chlorine compounds, *etc.*). Errors arising in the use of an IR pyrometer in a WTE boiler are mainly due to the flue-gas features and to environmental conditions [21,22].

#### 3.2.1. Errors Due to Gas Characteristics and Composition

The first error contribution is due to the fact that the flue-gas radiation proprieties are variable with both time and space and almost unknown. Knowledge of emission and absorption properties of the flue-gas are fundamental to regulate the IR pyrometer correctly, in order to perform an accurate temperature measurement [23]. The temperature error caused by lack of knowledge in radiance and emissivity can be estimated as:

(14) $$\Delta T_{m}=\frac{\lambda T^{2}}{c_{2}}\left(\frac{\Delta L_{\lambda ,m}}{L_{\lambda ,m}}-\frac{{\Delta \varepsilon }_{\lambda }}{\varepsilon_{\lambda }}\right)$$

where Δ*L*_λ_*_,m_* represents the difference between the measured and the true value of spectral radiance and Δε_λ_ represents the difference between the value of the instrumental emissivity and the true value, ε*_i_* − ε_λ_.

Equation (14) is general practice when the magnitudes of errors are known; if the errors are unknown, or perhaps random, their relationship to the measurement error is properly expressed in terms of uncertainty *u*:

(15) $$u(T_{m})=\frac{\lambda T^{2}}{c_{2}}\left(\frac{u_{L_{\lambda ,m}}^{2}}{L_{\lambda ,m}^{2}}-\frac{u_{\varepsilon_{\lambda }}^{2}}{\varepsilon_{\lambda }^{2}}\right)$$

The value of *c*_2_, one of the two constants of the Planck distribution law, is equal to 0.0143878 m·K.

Another source of error is fluorescence, which arises because thermal energy excites impurities in the gas (mainly silicon and carbon compounds), which then emit radiation in a very narrow band of wavelengths.

Scattering is an error due to the particles laden in the flue-gas. Smoke, luminous flames, water fog, carbon, metal or and silica in the flue-gas have three detrimental effects. Firstly particles scatter energy beyond the measurement volume, this causes a decrease in the measured radiance from the gas and therefore a decrease in the temperature reading. Secondly, particles may scatter radiation from other sources (mainly the flame) within the measurement volume, increasing the temperature reading. And thirdly, the particles may themselves emit blackbody radiation affecting the temperature measurement.

#### 3.2.2. Errors Due to Measurement Environment Conditions

All IR pyrometers display some sensitivity to the environmental conditions. The main sources of errors in this regard are given by reflections from flames, furnace and post-combustion membrane walls. In order to minimize these errors IR pyrometers have to be installed in proper places adjusting the shape and the length of the measurement volume and eventually shielding the thermometer detector from boundary interferences. Moreover, since energy radiated by a gas is low, a rather long response time has to be chosen.

In order to assure a stable measurement response, the IR pyrometer has to be cooled by a proper mass flow rate of demineralized water and the optical system has to be cleansed frequently. Both using a bare thermocouple and an IR pyrometer, the measured flue-gas temperature inside a WTE boiler differs, even by a number of degrees, from the true gas temperature.

Bare thermocouples are widely used because they are cheap and although the errors might reach more than 100 °C, their magnitude is quite stable after just a few hours of use. Moreover a bare thermocouple always measures a lower temperature than the true gas temperature.

IR pyrometers are more expensive than thermocouples and the errors that arise are usually smaller than those obtained with a bare thermocouple; on the other hand the temperature measured with a radiation pyrometer could be lower or higher than the one measured with a bare thermocouple. Although infrared pyrometers are increasingly being used, some operating engineers report that temperature measurements with these instruments give inaccurate results. They identify in the variability of the flue-gas composition, mainly due to dust content and chemical composition, the main source of errors.

**Figure 4.**
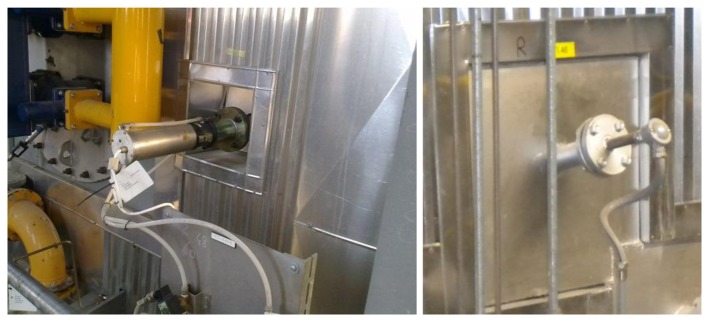
External views of an infrared radiation (IR) pyrometer on the left and of a bare thermocouple on the right, both installed on a WTE boiler.

Figure 4 shows an IR pyrometer and a bare thermocouple installed on a WTE boiler.

## 4. Temperature Measurements Using Suction Pyrometers

The design and operation of WTE steam generators depend on the evaluation of gas temperature in the furnace, in the post-combustion zone and in the superheater section of the boiler. The temperatures in the post-combustion zone are particularly important, both to meet the already cited European Directive and also to maximize the plant energetic efficiency.

A WTE boiler is always equipped with one or more auxiliary burners and these devices are turned on to ensure that boiler start-up and blow-out are correctly carried out, respecting functional parameters and limiting atmospheric emissions. The distributed control system (DCS) of the plant automatically turns on the burners, to prevent that the temperature in the post-combustion zone falls under 850 °C. Temperature readings at the DCS are supplied directly from the thermometers placed inside the post-combustion zone. An instrumental error deriving from these thermometers could therefore affect the energetic efficiency of the plant as a result of waste in fuel consumption, and harmful temperatures for the materials (refractory, *etc.*)

### 4.1. Suction Pyrometer Design and Measurement Technique

A suction pyrometer, also known as high velocity thermocouple (HVT), consists of a thermocouple protected from the chemical action of the flue-gas by an impermeable and thin coating of mineral oxide and placed in a system of low emissivity screens, which isolate the hot junction from the surrounding radiation.

The thermocouple is supported by a water cooled probe and the flue-gas is sucked at high velocity by a compressed air ejector through the screens and over the sheath. The gas velocity on the hot junction is adjustable by regulating the ejector pressure. Based on our experience the pressure value is decided upon at the beginning of the test and the velocity varies between 130 and 180 m/s, depending mainly on the inlet gas temperature.

The laboratory the leading author is in charge of has six suction pyrometers three of which are four meters in length and the other three are two meters in length. The thermocouples used in the pyrometers are calibrated by the same laboratory, which operates within the Italian Calibration Service.

**Figure 5.**
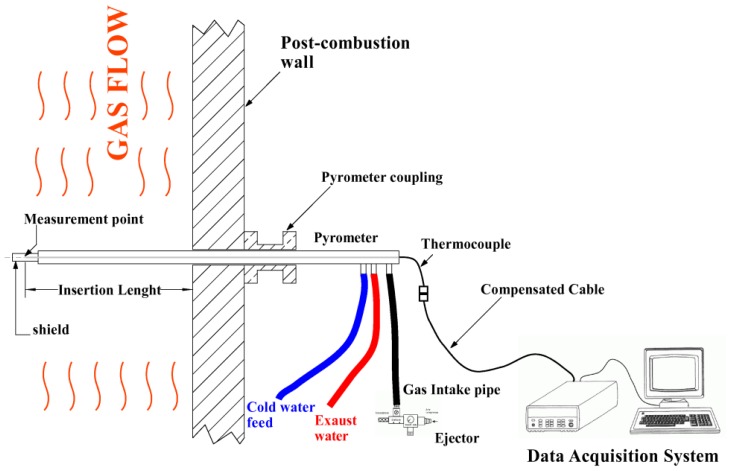
Lay out of a suction pyrometer with ancillary and data acquisition systems.

As shown in Figure 5, the measurement system is made up of a thermocouple, compensated extension cable and data acquisition system. Results and considerations presented in this work come from experience gained over a period of over ten years, in which the leading author has performed temperature measurements using one to six pyrometers simultaneously.

**Figure 6.**
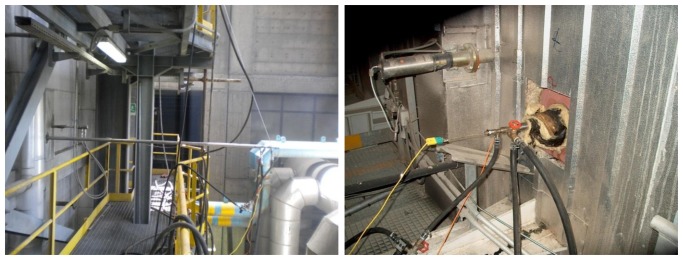
Suction pyrometer used for the *in situ* calibration of a thermocouple and an IR pyrometer.

On the left-hand side of Figure 6 a single suction pyrometer has been inserted alongside a bare thermocouple, with the same insertion length, whereas on the right-hand side of the figure the suction pyrometer has been inserted next to an IR pyrometer. This type of configuration enables *in-situ* thermometer calibration, for a given boiler thermal load.

**Figure 7.**
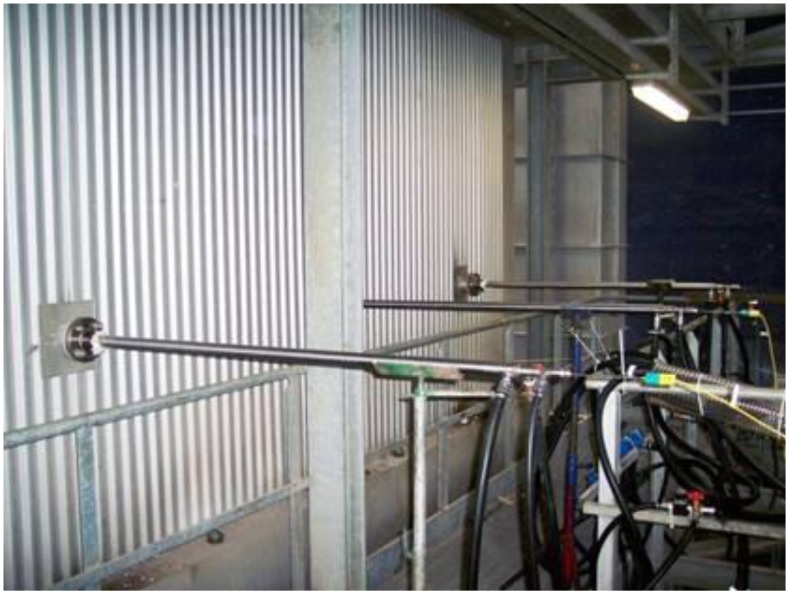
Three suction pyrometers are used to estimate the temperature profile in the post combustion zone of a WTE boiler.

In Figure 7 three pyrometers have been inserted at the same boiler ground level. This multiple pyrometer insertion, combined with a variable insertion length, allows estimation of the temperature profile in some characteristic zone of the incinerator plant. The multiple pyrometer insertion can also be vertical, which proves particularly useful when determining the vertical temperature gradient in different zones of the boiler.

The temperature readings obtained using the aforementioned methods are essential to satisfy different ends: in-situ calibration of thermometers, CFD model validation [24], the drawing up of mathematical algorithms for flue-gas resident time determination. To draw up the latter the gas mass flow rate inside the boiler needs to be known.

**Figure 8.**
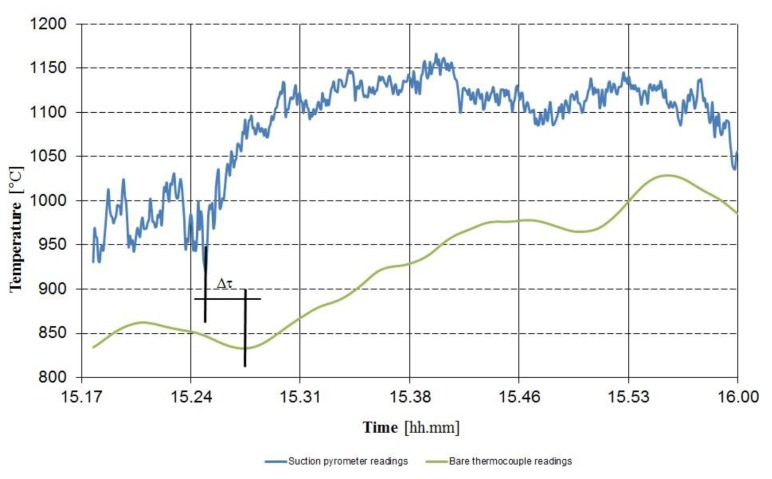
A comparison between suction pyrometers and thermocouple temperature readings in a WTE boiler under unsteady state conditions; a rough evaluation of the difference in the response times of the two thermometers is shown.

In Figure 8 a comparison between suction pyrometer and thermocouple temperature readings is reported.

### 4.2. Uncertainty Evaluation

This section analyzes the sources of error and the related uncertainties in the use of a suction pyrometer within a WTE boiler. The final result of this work will be the best estimation of the measurand and the estimation of its expanded uncertainty [25].

The measurand can be defined as the flue-gas temperature in a certain well-defined position inside the WTE boiler and in a defined boiler thermal load. The measurand, as it has been defined, will serve as a reference temperature for the in-situ calibration of bare thermocouples and IR pyrometers. For this reason it is indicated as *T_ref_*.

*T_ref_* evaluation takes into account a number of contributions: some of them related to measurement system and method, others to the conditions inside the measurement environment. Uncertainty related to the measurement system is herein indicated with *u_sys_*, whereas uncertainty related to the characteristics of the measurement environment with *u_env_*.

Uncertainty contributions that compound *u_sys_*, are related to: accuracy of the thermocouple used as a reference thermometer in the suction pyrometer and its stability, resolutions of the calibration system (suction pyrometer and data logger) and of the thermometers under calibration.

Uncertainty contributions that compound *u_env_*, take into account: repeatability (mean value and its deviation), the different displacement, inside the boiler, of the suction pyrometer and of the thermometer to be calibrated, effect of the environmental temperature on the data acquisition system.

Besides the mentioned statistical contributions, we also have one important systematic effect: it is related to the energetic balance at the thermocouple hot junction, see Equation (1).

#### 4.2.1. Systematic Contribution

The hot junction of the thermocouple inside a pyrometer receives heat by means of convection from the hot flue-gas and, at the same time, exchanges heat by radiation with the ceramic screens that, in turn, exchange heat by radiation with the boiler walls.

Under steady-state conditions and neglecting heat flux by conduction, the energetic balance at the hot junction of the thermocouple, inserted in the suction pyrometer, is simply:

(16) $${\dot{Q}}_{cv}={\dot{Q}}_{\text{rad}}$$

Heat flux by convection can be easily represented by means of the Newton equation:

(17) $${\dot{Q}}_{cv}=A_{cv}h_{cv}(T_{g}-T_{\text{pyr}})$$

where *A*_cv_ is the surface of the hot junction involved in the heat transfer, *h_cv_* is the convective heat transfer coefficient; *T_pyr_* and *T_g_* are respectively the equilibrium temperature of the junction and the flue-gas temperature.

Evaluation of the heat transfer coefficient is herein performed considering the calibration certificate of the ejector and [26]. The experimental results reported in the certificate of calibration of the ejector are shown in Table 1.

For each value of *P_ref_* a parameter *C* can be defined, so that:

(18) $$\dot{v}=C\cdot P_{\text{ref}}$$

The uncertainty of the volumetric flow rate of air sucked by the ejector can be estimated, for every value of *P_ref_* as:

(19) $$u\left(\dot{v}\right)=C\cdot u\left(P\right)$$

The uncertainty of pressure can be estimated dividing the expanded uncertainty *U*(*P*) by the coverage factor *k* = 2.

(20) u(P)=U(P)/k

The mass flow rate of air sucked by the ejector can be therefore calculated as:

(21) $$\dot{m}=\rho \cdot {\dot{v}}_{@30{}^{\circ}\text{C}}$$

The uncertainty of the mass flow rate is therefore:

(22) $$u\left(\dot{m}\right)=\rho \cdot u\left(\dot{v}\right)$$

Usually the pressure at the manometer of the ejector is fixed at 5.1 bar, so that the systematic error is corrected and the value of the mass flow rate is fixed.

Once the flue-gas mass flow rate is fixed, its velocity over the hot junction of the thermocouple is constant and equal to:

(23) $$w(T)=\frac{\dot{m}}{\rho (T)\cdot S}$$

where *S* is the inlet section of the suction pyrometer and ρ(*T*) is the flue-gas density.

The evaluation of the velocity uncertainty is performed fixing the flue-gas temperature, therefore:

(24) $$u\left(w\right)=\frac{u\left(\dot{m}\right)}{\rho \cdot S}$$

Reynolds number and its uncertainty can be estimated by the Equations (25) and (26):

(25) Re=ρ⋅w⋅dμ

(26) u(Re)=ρ(30°C)⋅dμ⋅u(w)

The Whitaker correlation (27) can be used to estimate the heat transfer coefficient by convection on spheres or cylinders. The frontal section of the hot junction of the thermocouple can in fact be assimilated to a sphere with a diameter *d*:

(27) Nu=2+(0.4Re0.5+0.06Re23)⋅Pr0.4⋅(μsμ∞)0.25

Pr is the flue-gas Prandtl number, μ*_s_* and μ_∞_ its dynamic viscosities respectively near the junction and in undisturbed conditions.

The evaluation of the Nusselt uncertainty can be performed attributing an intrinsic uncertainty *u_intr_* equal to 20% at the Whitaker correlation. Nusselt uncertainty can be therefore estimated through Equations (28)–(30):

(28) u(Nu)=(∂Nu∂Re)2⋅u2(Re)+uintr2(N\u)

(29) ∂Nu∂Re=[Pr0.4(μsμ∞)0.25]⋅(0.412Re+0.0623Re3)

(30) $$u_{\mathrm{int}r}^{2}\left(\text{Nu}\right)={\left(0.2\text{Nu}\right)}^{2}$$

The heat transfer coefficient and its uncertainty are then:

(31) $$h_{cv}=\frac{\text{Nu}\cdot \lambda }{d}$$

(32) $$u\left(h_{cv}\right)=\frac{\lambda }{d}\cdot u\left(\text{Nu}\right)$$

Heat flux by radiation is exchanged by the thermocouple junction and the ceramic screens of the pyrometer, which in turn exchange heat with the boiler walls. This flux can be calculated as:

(33) $${\dot{Q}}_{rd}=\frac{\sigma \cdot A_{rd}(T_{\text{pyr}}^{4}-T_{w}^{4})}{R_{rd}}$$

where *T_w_* is the surface temperature of the boiler walls, *R_rd_* is the thermal resistance by radiation and σ is the Stefan-Boltzmann constant.

The thermal resistance takes into account the total hemispherical emissivity of thermocouple (ε*_pyr_*), internal and external ceramic screens (ε*_is_* and ε*_es_*) and boiler walls (ε*_w_*). Its value can therefore be calculated as:

(34) $$R_{rd}=\frac{1-\varepsilon_{\text{pyr}}}{\varepsilon_{\text{pyr}}}+\frac{1}{F_{\text{pyr}-is}}+\frac{1-\varepsilon_{is}}{\varepsilon_{is}}+\frac{1-\varepsilon_{is}}{\varepsilon_{is}}+\frac{1}{F_{is-es}}+\frac{1-\varepsilon_{es}}{\varepsilon_{es}}+\frac{1-\varepsilon_{es}}{\varepsilon_{es}}+\frac{1}{F_{es-w}}+\frac{1-\varepsilon_{w}}{\varepsilon_{w}}$$

where *F* are the view factors between thermocouple and internal screen (*F_pyr−is_*), between internal screen and external one (*F_is-es_*) and between the external screen and the boiler wall (*F_es-w_*).

The thermocouple energetic balance can be now expressed as:

(35) $${\dot{Q}}_{cv}=h(T_{g}-T_{\text{pyr}})={\dot{Q}}_{rd}=\frac{\sigma \cdot (T_{\text{pyr}}^{4}-T_{w}^{4})}{R_{rd}}$$

The systematic contribution Δ*T_cv-rd_* is the ratio between the heat flux by radiation and the one by convection. It represents the difference between the gas temperature and the temperature measured with the suction pyrometers, as in the case of bare thermocouples this difference is always positive but its magnitude is lower:

(36) $$\Delta T_{cv-rd}=(T_{g}-T_{\text{pyr}})=\frac{{\dot{Q}}_{rd}}{h_{cv}}=\frac{\frac{\sigma \cdot (T_{\text{pyr}}^{4}-T_{w}^{4})}{R_{rd}}}{h_{cv}}$$

The uncertainty of the systematic effect is evaluated thus:

(37) $$u(\Delta T_{cv-rd})=\left(\frac{{\dot{Q}}_{rd}}{h_{cv}^{2}}\right)\cdot u(h_{cv})=\frac{\Delta T_{cv-rd}}{h_{cv}}\cdot u(h_{cv})$$

For each calibration condition, the systematic contribution and its expanded uncertainty are used to correct the temperature measured by the suction pyrometer, so that:

(38) $$T_{\text{ref}}=T_{\text{pyr}}+\Delta T_{cv-rd}-U(\Delta T_{cv-rd})$$

#### 4.2.2. Environmental Contributions *u_env_*

The value of *T_pyr_* is the arithmetic mean value of all readings obtained in the same measurement conditions: boiler thermal load and suction pyrometer displacement:

(39) $$T_{\text{pyr}}=\overset{n}{\underset{i=1}{\text{∑}}}T_{\text{pyr},i}/n$$

Its repeatability standard uncertainty can be assumed equal to the experimental standard deviation of the mean:

(40) $$u_{\text{rep}}(T_{\text{pyr}})=\sqrt{\frac{\overset{n}{\underset{i=1}{\text{∑}}}{\left(T_{\text{pyr},i}-T_{\text{pyr}}\right)}^{2}}{n\cdot (n-1)}}$$

Depending on the availability of inspection doors or test openings on the walls of the boiler, the suction pyrometer and the thermometer under calibration are placed as close as possible, but never occupy the same position within the boiler.

Due to the fact that the flue-gas temperature inside the boiler is not homogeneous, two additional uncertainty contributions, due to the different displacement of the thermometers inside the boiler, are to be taken into account.

The first contribution can be estimated performing a multiple pyrometer insertion inside the boiler, at two different ground levels. In this way the pyrometers are placed at a distance *H* and the thermometer under calibration is at an intermediate ground level. Using at least two pyrometers is firstly possible to calculate the vertical thermal gradient *GradT*, for a certain measurement condition. A typical value of this parameter ranges between 15 and 25 °C/m, depending on the surrounding wall composition (bare or coated with refractory).

Using the vertical thermal gradient is then possible to report the experimental mean value measured by the pyrometer $T_{\text{pyr}}^{H}$ at the same level of the thermometer under calibration. For each considered pyrometer placed at a distance Δ*H* from the thermometer under calibration:

(41) $$T_{\text{pyr}}=T_{\text{pyr}}^{H}\pm \text{GradT}\cdot \Delta H$$

The uncertainty contribution due to the temperature gradient can be estimated considering a rectangular distribution and attributing an error of 0,1 m in the estimation of Δ*H*:

(42) $$u_{\text{grad}}(T_{\text{pyr}})=\frac{\text{GradT}\cdot 0.1}{2\sqrt{3}}$$

A further uncertainty contribution is due to the different insertion length that the two instruments can have inside the boiler. This contribution can be estimated placing the suction pyrometer at different depths within the boiler. Temperature readings are taken in three different positions along the insertion length of the thermometer under calibration: at the same insertion length *L_lght_*, at *L_lght_* – 0.05 m and the last at *L_lght_* + 0.1 m.

This displacement uncertainty contribution *u_dis_* can be estimated considering a rectangular distribution and the difference Δ*T*_dis_ between the mean temperature values measured at *L_lght_* – 0.05 m and at *L_lght_* + 0.1 m:

(43) $$u_{\text{dis}}(T_{\text{pyr}})=\frac{\Delta T_{\text{dis}}}{2\sqrt{3}}$$

The effect of the ambient temperature on the data acquisition system can be determined, taking into account the technical specifications of the data logger and the mean value of the ambient temperature.

Considering a rectangular distribution, the uncertainty contribution due to the ambient temperature can be estimated as:

(44) $$u_{\text{ambT}}(T_{\text{pyr}})=\frac{1.0+0.03\cdot \left(T_{\text{amb}}-28\right)}{\sqrt{3}}$$

The environmental uncertainty contribution can be estimated as:

(45) $$u_{\text{env}}(T_{\text{pyr}})=\sqrt{u_{\text{rep}}^{2}(T_{\text{pyr}})+u_{\text{dis}}^{2}(T_{\text{pyr}})+u_{\text{grad}}^{2}(T_{\text{pyr}})+u_{\text{ambT}}^{2}(T_{\text{pyr}})+u_{cv-rd}^{2}(T_{\text{pyr}})}$$

#### 4.2.3. System Contributions u_sys_

The measuring chain of the suction pyrometer is calibrated before and after each measurement, temperature data of the calibration certificate are used to obtain two calibration curves. The first one is used to correct the mean value of the temperature *T_pyr_* measured by the pyrometer in a specified condition. The expanded uncertainty *U_cal_* on to the calibration certificate is used to obtain the instrument standard uncertainty of the measuring chain:

(46) $$u_{\text{cal}}(T_{\text{pyr}})=\frac{U_{\text{cal}}\left(T_{\text{pyr}}\right)}{\sigma }$$

The stability standard uncertainty of the measuring chain is then evaluated comparing the two calibration curves; for a selected value of *T_pyr_* the temperature difference Δ*T_cal_* obtained using the two calibration curves and a rectangular distribution are considered:

(47) $$u_{\text{stab}}(T_{\text{pyr}})=\frac{\Delta T_{\text{cal}}}{2\sqrt{3}}$$

Uncertainty related to the resolution of the pyrometer data acquisition system *u_pyr_* is evaluated considering the resolution *T_res-pyr_* and a rectangular distribution:

(48) $$u_{\text{pyr}}(T_{\text{pyr}})=\frac{T_{\text{res}-\text{pyr}}}{\sqrt{3}}$$

Uncertainty related to the resolution of the plant distributed control system (DCS) is evaluated in the same way, so:

(49) $$u_{\text{DCS}}(T_{\text{pyr}})=\frac{T_{\text{res}-\text{DCS}}}{\sqrt{3}}$$

The system uncertainty contribution can be estimated as:

(50) $$u_{\text{sys}}(T_{\text{pyr}})=\sqrt{u_{\text{cal}}^{2}(T_{\text{pyr}})+u_{\text{stab}}^{2}(T_{\text{pyr}})+u_{\text{pyr}}^{2}(T_{\text{pyr}})+u_{\text{DCS}}^{2}(T_{\text{pyr}})}$$

#### 4.2.4. Combined Uncertainty and Its Budget

The combined standard measurement uncertainty can be evaluated taking into account the environmental and system contributions:

(51) $$u(T_{\text{pyr}})=\sqrt{u_{\text{env}}^{2}(T_{\text{pyr}})+u_{\text{sys}}^{2}(T_{\text{pyr}})}$$

A typical uncertainty budget for a temperature of 900 °C is summarized in Table 2.

The expanded combined standard measurement uncertainty *U*(*T_pyr_*) can be estimated using a coverage factor *k* = 2, giving a coverage probability of about 95%:

(52) $$U(T_{\text{pyr}})=k\cdot u(T_{\text{pyr}})$$

## 5. Experimental Validation of the Calibration Method

In order to validate the calibration method proposed in the previous Section 4, the calibration of a bare thermocouple used in the post combustion zone of a WTE plant has been considered. The results presented in this section have been therefore obtained applying the relations and the statistical approach showed in the previous section.

The considered WTE plant is composed by two identical boilers; each boiler is equipped with a reciprocating water-cooled combustion grid and able to produce about 25 t/h of superheated steam which is fed to the turbine at a pressure of 4.50 MPa and at the temperature of 400 °C. The overall height of both the steam generators is about twenty-eight meters as referred to the ground level; their configuration is like those presented in Figure 1, with a radiant zone made by three vertical channels and a convective horizontal zone.

Six suction pyrometers have been used to calibrate different bare thermocouples installed in the post combustion zone of the considered boiler; for the sake of brevity and clarity the result of only one of these calibrations is here presented.

The bare thermocouple that has been calibrated is mounted in the front side of the first vertical channel of the boiler, at a level of 19.45 m over the ground and at 5.3 m above the last injection of air; its insertion length inside the post combustion chamber is of 0.6 m from the inner wall. Since no test openings are available at same level of the thermocouple, two suction pyrometers have been used for its calibration. Both the pyrometers have been inserted in the front side of the boiler, respectively at a height of 18.00 m and 26.5 m over the ground level, with the same insertion length of the thermocouple. The distance Δ*H*_1−2_ between the two insertion openings is therefore of 8.5 m.

**Figure 9.**
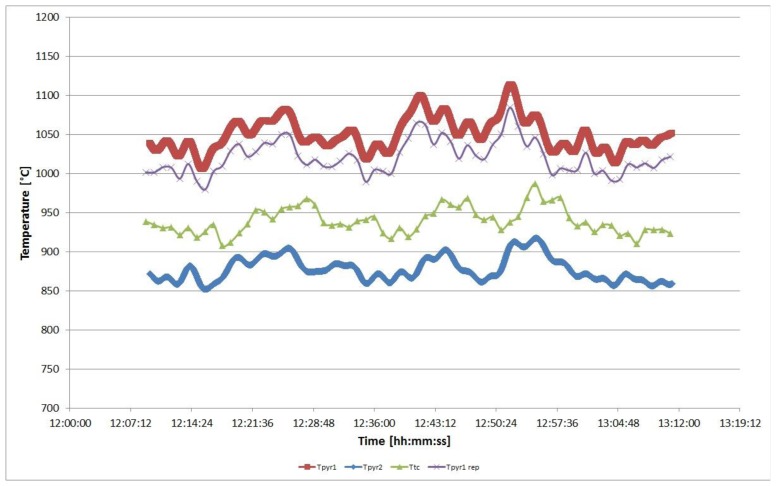
Temperature measured during the calibration by pyrometer 1, pyrometer 2 and by the bare thermocouple.

In Figure 9 the temperatures measured by the two suction pyrometers and by the bare thermocouple are represented: *T_pyr_*_1_ is the temperature measured by the lower pyrometer, whereas *T_pyr_*_2_ is the temperature measured by the pyrometer placed at 26.5 m, *T_tc_* is the temperature measured by the bare thermocouple. *T_pyr_*_1_*_rep_* is the temperature measured by the pyrometer 1, reported at the same level of the bare thermocouple by using Equation (41) and the value of the vertical gradient *GradT*, the latter calculated by the relation:

(53) $$\text{GradT}=\frac{T_{\text{pyr}1}-T_{\text{pyr}2}}{\Delta H_{1-2}}$$

In the previous equation *T_pyr_*_1_ and *T_pyr_*_2_ are the two mean values of the temperature readings of pyrometer 1 and 2 respectively. In this case *T_pyr_*_1_ is equal to 1049.59 °C, as shown by next Equation (54), whereas *T_pyr_*_2_ is equal to 877.61 °C. Since the pyrometer 1 is the closest to the thermocouple under calibration, its temperature readings have been manipulated in order to obtain the reference temperature for the thermocouple calibration.

The mean value of all temperature readings measured by pyrometer 1 and its repeatability standard uncertainty have been calculated by Equations (39) and (40), the numerical results are:

(54) $$\begin{array}{ll} T_{\text{pyy}1}^{H}=1049.59{}^{\circ}\text{C} & u_{\text{rep}}(T_{\text{pyr}1})=0.35{}^{\circ}\text{C} \end{array}$$

The systematic contribution and its expanded uncertainty have been calculated as described in Section 4.2.1, in this case the value of the systematic effect is Δ*T_cv-rd_* = 6.32 °C with an expanded uncertainty *U*(Δ*T_cv-rd_*) = 1.26 °C. Equation (38) allows therefore the calculation of *T_ref_* as:

(55) $$T_{\text{ref}}=1049.59+6.32-1.26=1054.65{}^{\circ}\text{C}$$

*T_ref_* is then corrected using the value of the vertical thermal gradient between pyrometer 1 and 2 in the considered conditions and the distance Δ*H* between pyrometer 1 and the thermocouple. Since the value of the temperature gradient *GradT* is equal to 20.23 °C/m and Δ*H* is equal to 1.45 m, Equation (41) gives:

(56) $$T_{\text{pyr}1}=1025.32{}^{\circ}\text{C}$$

The environmental uncertainty contributions are determined as described in Section 4.2.2, their numerical values are summarized in Table 3. In the same table the value of overall environmental uncertainty *u_env_*(*T_pyr_*), as determined by Equation (45) is reported.

The uncertainty contribution due to ambient temperature, *u_ambT_*, has been determined considering the mean value of the ambient temperature during the calibration process. In the presented case this value is *T_amb_* = 32.5 °C.

The system uncertainty contributions are determined as described in Section 4.2.3, their numerical values are summarized in Table 4. In the same table the value of overall system uncertainty *u_sys_*(*T_pyr_*), as determined by Equation (50) is reported.

The expanded combined standard uncertainty *U*(*T_pyr_*_1_) can be evaluated by considering Equations (51) and (52), the numerical results of these equations are respectively:

(57) $$u(T_{\text{pyr}1})=1.665{}^{\circ}\text{C and}\;U(T_{\text{pyr}1})=3.33{}^{\circ}\text{C}$$

The result of the thermocouple calibration is shown in Table 5, it clearly demonstrates that the value of the expanded uncertainty is very low, especially if compared with the temperature difference between the corrected mean value given by the suction pyrometer and that given by the bare thermocouple.

## 6. Conclusions

The present work shows that the use of suction pyrometers in WTE boilers allows particularly accurate temperature measurements compared to those obtained through the use of conventional thermocouples or optical pyrometers, normally installed on such systems.

The measurement campaign with suction pyrometers on a WTE plant must be properly planned and its realization may take a few days. Particularly important are: the choice of the test openings for measurement among those available on the steam generator, the choice of the insertion length of the instruments i nside the generator, the thermal loads to be investigated, the duration of the test and the choice of the sampling frequency.

The temperature measurements obtained, generally have a low measurement uncertainty and the best results in this direction are obtained and measured for a period of at least twenty minutes and with a constant boiler thermal load.

The results of the measurements carried out with suction pyrometers, allow the *in-situ* calibration of the plant thermometers (IR thermocouples or pyrometers), used by the DCS for the continuous temperature measurement. In a properly designed plant in which in-situ calibrated thermometers are installed, the interventions of the auxiliary burners are limited to the stages of switching on and off of the plant.

Under these conditions, based on experience, valuable fuel savings can be up to 50%, in addition, the coating materials (refractory) and system components are subjected to less thermal stress. For this reason also the frequency of unscheduled shutdowns, for the execution of extraordinary maintenance, is greatly reduced.

## Figures and Tables

**Table 1.** Results of the ejector calibration.

***P****_ref_***(bar)**	***P****_ind_***(bar)**	***P****_ref_* − ***P****_ind_***(bar)**	***U*(*P*) (bar)**	*ν̇* @**30** °**C (m^3^/s)**
0.0000	0.0	0.0	0.06	0.0
1.0000	1.1	0.1	0.06	–
2.0000	2.1	0.1	0.06	1.5277 × 10^−3^
3.0000	3.1	0.1	0.06	2.0833 × 10^−3^
4.0000	4.1	0.1	0.06	2.3611 × 10^−3^
5.0000	5.1	0.1	0.06	2.5000 × 10^−3^
6.0000	6.2	0.2	0.06	2.5960 × 10^−3^

**Table 2.** Typical uncertainty budget for *T_pyr_* = 900 °C.

**Contribution**	**Symbol**	**Distribution**	**Typical value** (°**C)**
Repeatability	*u_rep_*	normal	1.00
Vertical gradient	*u_grad_*	rectangular	0.70
Insertion length	*u_dis_*	rectangular	1.00
Ambient temperature	*u_ambT_*	rectangular	0.78
Convective-radiative	*u_cv-rd_*	normal	1.00
Calibration	*u_cal_*	normal	0.70
Stability	*u_stab_*	rectangular	0.60
Pyrometer resolution	*u_pyr_*	rectangular	0.0033
DCS Resolution	*u_DCS_*	rectangular	0.033
	
Standard uncertainty *u*(*T_pyr_*) (°C)			2.22

**Table 3.** Environmental uncertainty contributions.

**Contribution**	**Symbol**	**Value (**°**C)**
Repeatability	*u_rep_*	0.35
Vertical gradient	*u_grad_*	0.58
Insertion length	*u_dis_*	0.50
Ambient temperature	*u_ambT_*	0.67
Convective-radiative	*u_cv-rd_*	0.63
Overall environmental	*u_env_*	1.25

**Table 4.** System uncertainty contributions.

**Contribution**	**Symbol**	**Value (**°**C)**
Calibration	*u_cal_*	0.70
Stability	*u_stab_*	0.22
Pyrometer resolution	*u_pyr_*	0.58
DCS resolution	*u_DCS_*	0.58
Overall system	*u_sys_*	1.10

**Table 5.** Results of the calibration.

**Reference temperature** ***T****_pyr_***_1_(**°**C)**	**Expanded uncertainty** ***U*(*T****_pyr_***_1_) (**°**C)**	**Thermocouple temperature** ***T****_tc_***(**°**C)**
1025.32	3.33	938.60
